# Exploring the potential of biphenylamine- and triphenylamine-based sensitizers for enhanced efficiency of more than 8% in dye-sensitized solar cells

**DOI:** 10.1098/rsos.240133

**Published:** 2025-10-29

**Authors:** Faoz H. Al-Atawi, Ahmad Irfan, Abdullah G. Al-Sehemi

**Affiliations:** ^1^ Department of Chemistry, College of Science, King Khalid University, P.O. Box 9004, Abha 61413, Saudi Arabia

**Keywords:** dye-sensitized solar cells, triphenylamine, light-harvesting efficiency, adsorption behaviour, absorption spectra, power conversion efficiency

## Abstract

As the world’s population and industrial sector rapidly expand, the demand for energy is rising. Solar cells can address both global energy and environmental needs. With the aim to enhance dye-sensitized solar cell (DSSC) efficiency, we designed four metal-free biphenylamine- and triphenylamine-based dyes (RK2–RK5) based on the reference dye RK1, increasing the donor strength (substituting different groups such as biphenylamine and triphenylamine into the donor side of the dye) as such dyes improved DSSC power conversion efficiency. Density functional theory (DFT) was applied at the B3LYP/6–31G** level to calculate the ground-state (S_0_) optimized geometries of RK1-RK5. Time-dependent DFT was used to compute the absorption spectra, utilizing four functionals (B3LYP, CAM-B3LYP, PBE1PBE and BHandHLYP), in the gas phase and in solvents such as dichloromethane and ethanol. Comprehensive analysis of RK1-RK5 as well as dyes@TiO_2_ was performed, and light was shed on the optoelectronic properties. Frontier molecular orbitals' charge density distribution revealed the sensitizers' intramolecular charge transfer from the donor to the acceptor unit. After adsorption of the dyes on TiO_2_, charge transfer was seen from sensitizer to the TiO_2_ semiconductor’s surface in dyes@TiO_2_. Adsorption of the dyes on the TiO_2_ cluster would be stable, as revealed by the dyes@TiO_2_ cluster’s negative binding energy. Additionally, it was found that the presence of two donor groups raises the electronic coupling and electron injection constants in RK4 and RK5, indicating that the charge injection in these newly designed dyes would be superior. As a result, the DSSC efficiency in the newly designed derivatives has been improved to 8.05% for RK5 by substituting the triphenylamine unit at the R1 and R2 positions in the parent compound. These well established correlations between structure–property relationships and performance provide profound insight into how improving the donor moiety strength in organic sensitizers affects device performance. It boosted photovoltaic performance through enhanced short-circuit current density and light-harvesting efficiency. For high efficiency in DSSCs, this can provide a useful rational molecular design strategy for D–π–A organic sensitizers.

## 1. Introduction

To prevent environmental pollution and rising prices, sources of non-renewable energy must be substituted with sources of renewable energy to avoid the disadvantages of non-renewable energy sources. Geothermal energy, wind, sunlight, rain and tides are all sources of renewable energy. Sunlight, which is employed in a variety of ways in our daily lives, is one of the main sources of renewable energy [1]. It is one of the world’s most abundant, cleanest and renewable energy sources, and its use is rapidly expanding. The amount of energy that the Sun produces and radiates into space is far greater than that which can be utilized for electricity by humankind. The Sun radiates energy regularly in all directions at a rate of 400 billion quadrillion (4 × 10^26^) watts. The Earth captures a small fraction of this energy: 170 quadrillion (1.7 × 10^17^) watts, or roughly half a billionth of it [2]. In order to meet the energy demands due to the increasing population and avoid the pollution coming from non-renewable energy, energy conversion from sunlight is a sustainable method to produce energy in the long term [3]. Currently, one of the most popular solar energy techniques for electricity production is the solar cell [4]. In a solar cell, p- and n-type semiconductor materials are sandwiched to create at least two layers [5]. An n-type semiconductor typically has an overabundance of electrons, whereas a p-type semiconductor typically has an overabundance of holes [6]. Solar cells' efficiency can range between 6% for amorphous silicon-based solar cells up to 44.0% for multiple-junction cells, increasing to 44.4% with several dyes combined into a hybrid. Regarding multicrystalline Si solar cells that are offered commercially, their energy conversion efficiency ranges from 14 to 19%. Not all cells with high efficiency are cost-effective. For instance, a low-volume, 30% efficient multi-junction cell made of exotic compounds like indium selenide or gallium arsenide may cost a hundred times as much as a mass-produced, 8% effective amorphous silicon cell, though the latter would only produce four times less output [7–9].

One efficient solar cell technology is dye-sensitized solar cells (DSSCs). The DSSC was identified as a Gratzel solar cell [10]. The DSSC is a good replacement for traditional inorganic, silicon solar cells [11]. The inexpensive cost of production and low purification requirements of DSSCs make them advantageous and attractive [10]. The most important five components of DSSCs are a dye, a semiconductor oxide, a counter-electrode, an electrolyte and a transparent conducting glass substrate [12]. There are some requirements for maintaining the flow of electrons when sunlight is absorbed by the dye in DSSCs. The dye must be adsorbed on a semiconductor that has a wide band gap. For example, the lowest unoccupied molecular orbital (LUMO) level of the dye must be higher than the conduction band (CB) of the semiconductor oxide to inject the photoexcited electrons easily into the CB of the semiconductor, leaving the dye in its oxidized state [13]. Furthermore, the dye’s highest occupied molecular orbital (HOMO) level must be lower compared with that of the electrolyte. The dye is then easily regenerated by transferring an electron from the electrolyte. The electrolyte typically used in DSSCs is the iodide/triiodide redox system. Device components interact complexly, especially at the semiconductor oxide/sensitizer/electrolyte interface. However, environmental factors like temperature, solar radiation, and operating conditions also influence these interactions [14].

**Figure 1.**
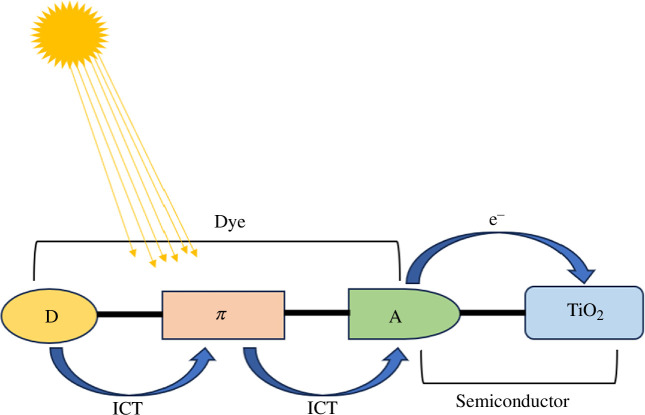
Schematic view of the donor–π-bridge–acceptor principal structure of dye with TiO_2_ photoanodes in DSSCs.

**Figure 2.**
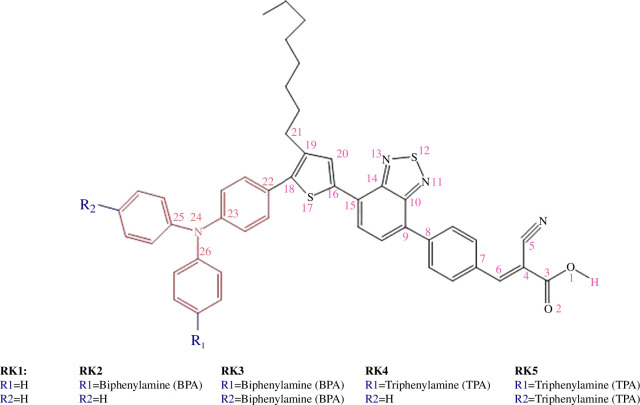
Structures of RK1 and its derivatives RK2–RK5.

**Figure 3.**
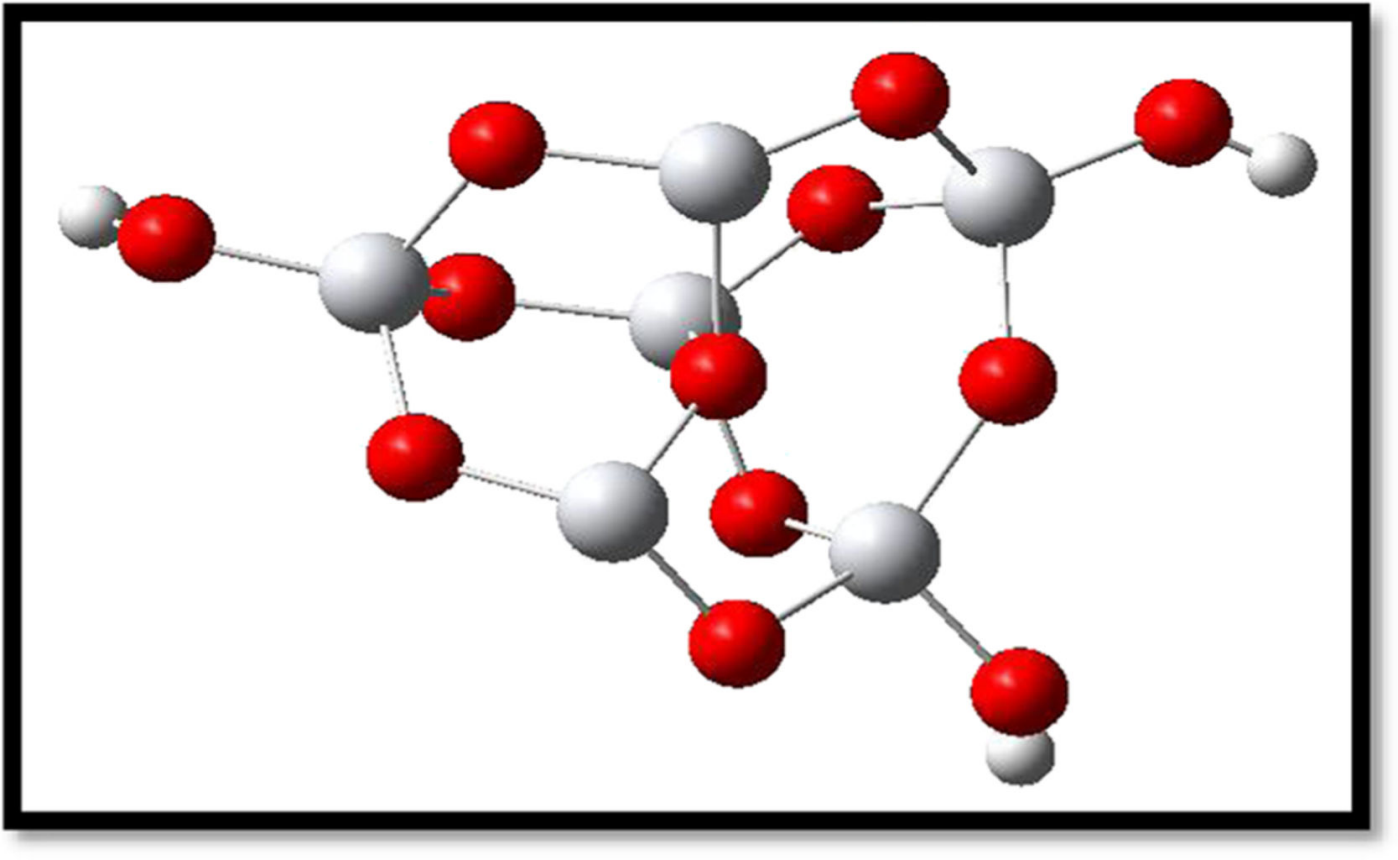
Structure of Ti_6_O_12_H_3_ cluster.

The dye is one of the most important elements of a DSSC. So, the consideration of optoelectronic and electronic properties is crucial in the molecular design of dyes for DSSCs [12,15]. Researchers have designed plenty of metal-free organic dyes for use in DSSCs and successfully enhanced the power conversion efficiency [16–21]. Some examples of donor units include triphenylamine (TPA) [22–29], coumarin [30–33], indole [34–36], phenothiazine [37–40], carbazole [39,41,42], squarine [43,44] and cyanine [45–47]. A promising dye for established DSSC applications is a TPA-based dye (see electronic supplementary material, figure S1) [42]. It is an excellent candidate for DSSC applications because of its important advantages, which include higher stability, an electron-donating capacity, and a nonplanar molecular configuration that is resistant to aggregation [48–53]. Reduced aggregation of the TPA-based dye facilitates the injection of electrons into the semiconductor’s CB from the excited dye. So too, the recombination of injected electrons with the electrolyte can be inhibited by the propeller-shaped TPA architecture [48]. Also, charge transport, acceptable ionization potentials (IPs), and superior thermal and morphological stability have all been demonstrated by the TPA-based hole transport materials (HTMs). The TPA-based oligomer molecules come in a variety of shapes, such as linear, spiro and starburst structures. The efficiency of DSSCs using TPA as a donor and various spacers was determined to be 5.3, 7.33 and 9.8% [54]. In another study, the efficiency of DSSCs using TPA-based organic dye achieved about 10 [55], 14 [56] and over 15% by co-sensitization [29]. DSSCs have confirmed yields of about 14.3%, exceptional endurance over 20 000 h of continuous exposure, and years of extrinsic experimentation [57]. The most commonly used design for organic dyes is a push–pull strategy, represented as D–π–A, where D stands for the electron donor unit, π for the bridge spacer and A for the electron acceptor moiety (see figure 1). The electron acceptor moiety is anchored on the surface of the semiconductor [58,59]. Since cyanoacrylic acid (C_4_H_3_NO_2_) is an effective electron-withdrawing unit and firmly anchors on the surface of the semiconductor, we used it as the acceptor unit in designing targeted dyes for application in DSSCs [60]. Based on prior research indicating that the thiophene moiety is the best π-spacer owing to its increased light-harvesting efficiency (LHE), we employed thiophene units as π-spacer in our designed dyes [61]. This work aimed to comprehend the influence of increasing the strength of the donor unit in the metal-free TPA-based dyes to boost the performance of the DSSC devices (see figure 2). We studied the effect of donor strength on the optoelectronic characteristics and efficiency of studied dyes. We selected dye RK1 because it has inexpensive precursors, and it produced good power conversion efficiency [62]. Haid *et al*. demonstrated that introducing a phenyl ring between the anchoring benzothiadiazole (BTD) and the cyanoacrylic acid unit stabilizes the dye radical cation and reduces the recombination rate [63]. Moreover, the interaction surface between the sensitizers RK1-RK5 and the TiO_2_ cluster during the adsorption of the sensitizer results in a medium-sized (Ti_6_O_12_H_3_) cluster (see figure 3).

## 2. Methodology

### 2.1. Validation of the adopted methodology

The optoelectronic and charge transport characteristics of all the dyes were effectively predicted by quantum chemical approaches. Advancements in density functional methods have contributed to their exceptional predictive power in the computation of electronic, charge transport and optical characteristics. The DFT calculations were accomplished with the Gaussian 16 program at the B3LYP functional and 6–31G** basis set to optimize ground-state (S_0_) geometry calculations of all RK1–RK5 dyes, as displayed in electronic supplementary material, figure S2.

To validate the employed methodology, we examined the RK1 dye’s properties [62] (see electronic supplementary material, figure S3), and then carried out an exhaustive examination to increase the accuracy of the results. Moreover, strengthening the donor part within our designed dyes (RK2–RK5) would be a good way to improve the charge transport properties. Electronic supplementary material, figure S2 displays the optimized structures of RK1–RK5. The experimental outcome was compared with the optimized geometrical parameters, which included bond lengths, bond angles and dihedral angles [62]. The optimized structure is trustworthy for additional study because of the strong correlation found between the computed and experimental parameters (see electronic supplementary material, figure S4), which further demonstrates the reasoning behind our chosen approach (details are provided in §3a).

Time-dependent DFT using four functionals, B3LYP, BHandHLYP, CAM-B3LYP and PBE1PBE, with the 6–31G** basis set in the gas phase, DCM solvent and ethanol solvent has been used to calculate the absorption spectra $\lambda_{\text{max}}$, since $\lambda_{\text{max}}$ was measured experimentally in the ethanol solvent. We calculated the value and checked the effect of the solvent on $\lambda_{\text{max}}$. The results acquired using the PBE1PBE functional with the 6–31G** basis set are quite close to the experimental value. So, the $\lambda_{\text{max}}$ values computed at DCM-PBE1PBE, that is, 464 nm and at Ethanol-PBE1PBE, that is, 463 nm, are in good agreement with the experimental result (470 nm) [62]. Thus, we adopted these functions to assess the $\lambda_{\text{max}}$ values of the designed derivatives and the dyes@TiO_2_ cluster. The results of the experiments were correlated with the computed energy gaps (*E*_gap_), which are the difference between the *E*_HOMO_ and *E*_LUMO_. Finally, the DSSC efficiency was computed (details are given in the electronic supplementary material).

## 3. Results and discussion

### 3.1. Ground-state optimized geometries

**Figure 4.**
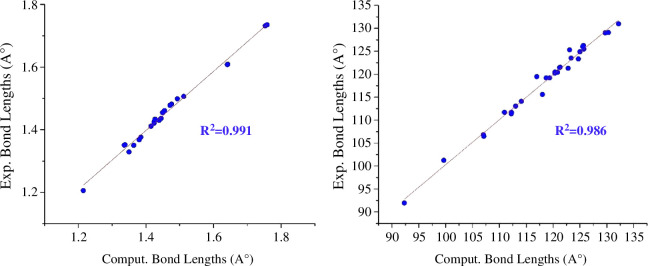
Correlation graphs of the bond lengths (Å) (left) and bond angles (°) (right) of isolated dye RK1 between experimental values and values computed at the B3LYP/6–31G** level.

Our primary reason for characterization and synthesis using computational methods is the molecular design of the new dyes. Thus, by investigating new DSSC devices, experimentalists might gain significant insight into the synthesis of efficient dyes. Previous studies have mostly concentrated on the structure, optoelectronic, and charge transfer characteristics of organic sensitizers. Figure 1 depicts the architectural design structure of the current sensitizers studied. The organic dye RK1, synthesized by Joly *et al*. [62], then utilized in DSSC devices, was chosen as the reference dye in this present study. Figure 2 shows our designed structures for the sensitizers RK2–RK5. Figure 4 displays the correlation graphs between selected bond lengths (Å) and bond angles (°) of the isolated dye RK1 between the experimental results and those calculated at the B3LYP/6–31G** level.

The computed geometrical parameters for RK1 are displayed in detail in electronic supplementary material, tables S1–S3, and they agree well with the experiment results [62]. Additionally, the bond lengths, bond angles and dihedral angles of RK2–RK5 are listed in electronic supplementary material, tables S4–S15. Selected bond angles and dihedral angles of the isolated sensitizers RK1–RK5 are shown in table 1. The bond angle changes from neutral to cation for RK1–RK5 are 0.9, 0.59, 0.48, 0.55 and 0.41^o^, while from neutral to anion they are 2.45, 2.42, 2.39, 2.41 and 2.39^o^, respectively. The values obtained indicate that a bigger change from neutral to anion would result in increased polarization. The reorganization energy rises with increasing polarization. For RK1–RK5, the dihedral angle changes from neutral to anion are greater than those from neutral to cation, indicating more polarization in anionic species and a consequent increase in electron reorganization energy ($\lambda_{\text{e}}$). In RK2 and RK4, the changes in the dihedral angle C14–C15–C16–C20 from neutral to cation are 0.91 and 0.92^o^, respectively. This indicates that RK2 and RK4 may be the better materials because they have less polarization. Hence, the smaller the $\lambda_{\text{e}}$, the less polarization from neutral to cation (details can be found in §3.6). For example, the $\lambda_{\text{h}}$ for RK2 and RK4 is 0.14 and 0.21 eV, respectively, which is smaller than for RK1 because RK2 and RK4 have less polarization as a result of the BPA and TPA units substituting for the H at position R1 in the dye RK1. The bond angle O1–C3–O2 of RK1–RK5 increases from neutral to cation while decreasing from neutral to anion, but the polarization in anionic species is three-to-fourfold more than in cationic species.

**Table 1.** The bond and dihedral angles of isolated dyes RK1–RK5 at the B3LYP/6–31G** level.

	bond angle (°)									
	O1–C3–O2									
	RK1	RK2	RK3	RK4	RK5					
neutral	123.06	123.05	123.04	123.05	123.05					
cation	123.96	123.64	123.52	123.6	123.46					
Δ	0.9	0.59	0.48	0.55	0.41					
anion	120.61	120.63	120.65	120.64	120.66					
Δ	2.45	2.42	2.39	2.41	2.39					

	dihedral angle (°)									
	C14–C15–C16–C20					C14–C15–C16–S17				
	RK1	RK2	RK3	RK4	RK5	RK1	RK2	RK3	RK4	RK5
neutral	−5.86	−5.53	−5.71	−5.67	−6.16	174.12	174.41	174.27	174.33	173.82
cation	−4.36	−6.44	−8.50	−6.59	−8.33	175.14	172.99	170.94	172.92	171.16
Δ	1.5	0.91	2.79	0.92	2.17	1.02	1.42	3.33	1.41	2.66
anion	−1.18	−1.25	−1.38	−1.37	−1.52	178.36	178.35	178.19	178.15	178.03
Δ	4.68	4.28	4.33	4.3	4.64	4.24	3.94	3.92	3.82	4.21

### 3.2. Electronic properties

**Figure 5.**
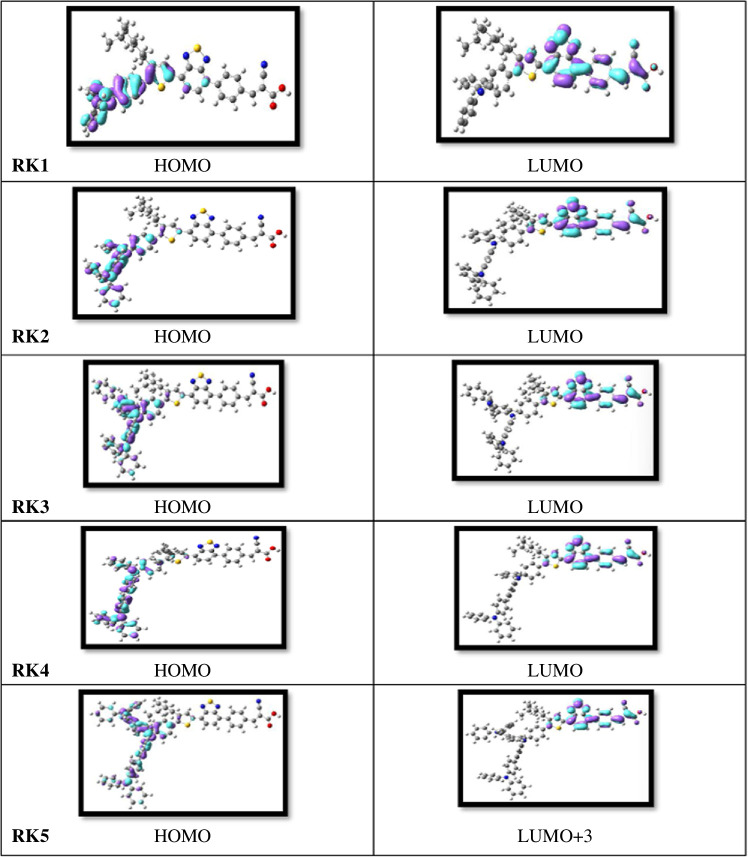
Distribution pattern of the frontier molecular orbitals of RK1–RK5 at the B3LYP/6–31G** level.

Understanding the characteristics of the charge transfer from donor (D) to acceptor (A) requires insight regarding charge distribution and energy levels of the HOMOs and LUMOs. In this work, the influence of substituting group TPA or BPA as donor unit of the designed sensitizers is discussed. The charge density of the frontier molecular orbitals (FMOs), and the LUMOs and HOMOs of isolated dyes and adsorbed dyes (dyes@Ti_6_O_12_H_3_), of RK1–RK5 are illustrated in figure 5 and in electronic supplementary material, figures S5–S7.

The main transitions for isolated dyes RK1–RK4 are from HOMO to LUMO, but in RK5 they are from HOMO−2 to LUMO + 3, contributing to the charge transport (see figure 5). The two major transitions for RK1 and RK2 are the HOMO → LUMO and HOMO−1 → LUMO, while the major transitions for the RK3 are the HOMO → LUMO and HOMO−2 → LUMO, contributing to the charge transport (see figure 5 and electronic supplementary material, S5). The major transitions for RK4 are HOMO → LUMO and HOMO−1 → LUMO, while the major and best transition for the RK5 is the HOMO → LUMO + 3, contributing to the charge transport (see figure 5 and electronic supplementary material, figure S5). Also, the BPA group makes a good contribution to the HOMOs in RK2 and RK3 (see figure 5). The density distribution behaviours and transitions of these dyes look like those of the RK1 dye. The HOMO and HOMO–1 in RK4, which is mono-substituted with TPA, are also delocalized on the donor side and cover the TPA after replacing the hydrogen at R1 in RK1 (see figure 5 and electronic supplementary material, figure S5). However, the HOMO and HOMO−2 in RK5, which is di-substituted with TPA, are delocalized on the donor moiety and do not much cover the TPA replacing the hydrogens at positions R1 and R2 in RK1 (see electronic supplementary material, figure S5). Figure 5 In the illustration, the HOMO is delocalized at the TPA moiety, and the LUMO mainly at thiophene and the acceptor side. The HOMO density has been delocalized on the central ring and/or donor side while LUMO is localized on the thiophene and acceptor side.

Additionally, the best transitions for the dyes RK1–RK5 after adsorption on the TiO_2_ cluster are illustrated in detail in electronic supplementary material, figures S6 and S7. However, the main transition, for RK1@Ti_6_O_12_H_3_ from HOMO−2 → LUMO+1 level, for RK2–RK4@Ti_6_O_12_H_3_ from HOMO → LUMO+1 level, and for RK5@Ti_6_O_12_H_3_ from HOMO−3 → LUMO+1, contributes to the charge transport. In the precise depiction, the HOMO is delocalized on the TPA moiety, and LUMO+1 mainly at the acceptor and the TiO_2_ cluster. For the RK1@TiO_2_ and the RK5@TiO_2_ clusters,HOMO−2 and HOMO−3 are delocalized on the TPA moiety and the centre of this sensitizer, while the HOMO is delocalized on the donor side of RK2@TiO_2_, RK3@TiO_2_ and RK4@TiO_2_ clusters (see electronic supplementary material, figure S7). The LUMO is seen at the acceptor side but mostly on the TiO_2_ cluster (see electronic supplementary material, figure S7).

However, the TPA and BPA groups contribute to the HOMO when they are substituted at position R1 and/or R2 in RK1. The TPA and BPA units have been introduced by replacing the hydrogens of two aryls at R1 and/or R2 in RK1 (see figure 2).

Photophysical and electrochemical characteristics like luminescence should be present for the dyes. They should cover a wide range of ultraviolet–visible (UV–vis) wavelengths, the LUMO/HOMO of the dyes should lie above/below the CB of TiO_2_, and the redox electrolyte should be above the HOMO level (–4.60 eV for $I^{-}$/$I_{3}^{-}$) [64–66]. The calculated values of *E*_HOMO_, *E*_LUMO_ and *E*_gap_ are shown in table 2. The computed values of *E*_HOMO_/*E*_LUMO_ of the isolated parent dye are −5.07/−2.88 eV, respectively, as shown in table 2. The calculated *E*_HOMO_ and *E*_LUMO_ of the designed derivatives RK2, RK3, RK4 and RK5 are −5.36/–2.87, −5.15/–2.86, −4.94/–2.72 and −4.77/–2.87 eV, respectively and the *E*_gap_ value is 2.49, 2.29, 2.21 and 1.89 eV, respectively. In DSSCs, the dye’s *E*_gap_ value is fundamental. Among isolated sensitizers, RK5 has the lowest *E*_gap_, that is, 1.89 eV, because of two donor TPA moieties at R1 and R2, as illustrated in electronic supplementary material, figure S8. Also, the mono-substitution of a TPA moiety at position R1 in RK1 gives RK4 a lower *E*_gap_, that is*,* 2.21 eV, than the *E*_gap_ values of RK2 and RK3. Among the isolated dyes, RK2 has the lowest *E*_HOMO_ but RK1 has the lowest *E*_LUMO_ level. The best dyes are RK5 > RK1 > RK4 > RK3 > RK2, in that order, based on the lower value of the *E*_gap_. After adsorption of the RK1–RK5 dyes on the TiO_2_ cluster surface, the energy of the LUMO levels is lower than that of the isolated sensitizers RK1–RK5. Moreover, after adsorption of the RK1 and RK5 dyes on the TiO_2_ surface, the *E*_HOMO_ levels are mainly lower than in the isolated sensitizers RK1 and RK5, but in RK2–RK4 the *E*_HOMO_ levels are significantly higher than in the isolated counterparts. Also, after adsorption of the RK1–RK5 on the TiO_2_ surface, the *E*_gap_ is somewhat lower than in the isolated sensitizers RK1–RK5. RK3 has the lowest *E*_gap_ as compared with RK1, RK2, RK5 and RK4 owing to the increase in the strength of the donor groups by replacing the hydrogen atoms with BPA units at both positions R1 and R2 in the RK1 dye. Also, when we move from the RK1@TiO_2_ to the RK5@TiO_2_ cluster, we notice that the RK3@TiO_2_ cluster has the lowest energy gap of all owing to having two BPA units in the donor moiety, substituted at both positions R1 and R2 in the RK1 dye. Moreover, the RK5@TiO_2_, RK4@TiO_2_ and RK2@TiO_2_ clusters have smaller *E*_gap_ values than the RK1@TiO_2_ cluster. As a result, increasing the strength of the donor unit, particularly by substituting two TPA or two BPA units for the donor groups of RK1, had an impact on *E*_HOMO_, *E*_LUMO_ and *E*_gap_. According to table 2, the dyes with the lowest *E*_gap_ value are RK3 > RK5 > RK2 > RK4 > RK1, in that order.

**Table 2.** The *E*_HOMO_, *E*_LUMO_ and *E*_gap_ at the B3LYP/6–31G** level of theory for parent molecule RK1, designed molecules RK2–RK5, and dyes@Ti_6_O_12_H_3_.

	*E*_HOMO_ (eV)	*E*_LUMO_ (eV)	*E*_gap_ (eV)
RK1	−5.07	−2.88	2.19^a^
RK1@TiO_2_ cluster	−5.09	−3.41	1.67
RK2	−5.36	−2.87	2.49
RK2@TiO_2_ cluster	−4.89	−3.39	1.50
RK3	−5.15	−2.86	2.29
RK3@TiO_2_ cluster	−4.74	−3.39	1.35
RK4	−4.94	−2.88	1.98
RK4@TiO_2_ cluster	−4.92	−3.40	1.52
RK5	−4.77	−2.87	1.89
RK5@TiO_2_ cluster	−4.84	−3.40	1.44

^a^ Experimental *E*_gap_ is 1.65 eV [62].

Moreover, the HOMO levels of RK1–RK5 are −5.07, −5.36, −5.15, −4.94 and −4.77 eV respectively, lower than the value for the electrolyte $I^{-}$/ $I_{3}^{-}$ (−4.60 eV), demonstrating that the oxidized dyes can be easily regenerated by the $I^{-}$/ $I_{3}^{-}$ during the process of sensitizer regeneration. Also, the LUMO levels should be higher than the CB edge of the TiO_2_ (−3.74 eV) [67]. The LUMO energies of RK1–RK5 are −2.88, −2.87, −2.86, −2.72 and −2.87 eV, respectively, higher than the CB edge of the TiO_2_, ensuring that the excited state dye electrons may be sufficiently injected into the CB of the TiO_2_ semiconductor during the charge injection operation. The energy level diagram of RK1–RK5 is shown in electronic supplementary material, figure S8.

### 3.3. Molecular electrostatic potential

The molecular electrostatic potential (MEP) can be assessed for various materials using diffraction spectra and/or computational approaches. Various colours can be used to illustrate the wide range of electronic and nuclear changes. Blue < green < yellow < orange < red is the order in which the negative MEP increases. Furthermore, MEP locates the location of the most reactive region inside the molecule, furnishes details regarding the molecule’s stability and identifies the zone that possesses the most negative or positive potential, respectively favouring an electrophilic or nucleophilic substitution attack. Given a greater negative area, the molecule may be a more photostable compound. Additionally, MEP are useful in determining various interactions between molecules. The colours of the MEP-mapped surfaces are displayed in electronic supplementary material, figures S9 and S10. There, the higher negative potential zones are shown in red. This colour indicates that electrophilic substitution is favoured. On the other hand, the higher positive potential zones are indicated by blue, and are advantageous for nucleophilic substitution attack. On the acceptor group and semiconductor surface, a negative electrostatic potential was discovered, but a positive one is observed at the acceptor group’s hydrogen atoms and a little bit along the elongating side chains, e.g. RK1–RK5 exhibit higher negative electrostatic potential on the acceptor part as well as the entire systems except for the donor part (see electronic supplementary material, figure S9) [68]. The adsorbed dyes on the Ti_6_O_12_H_3_ surface showed negative potential zones mostly located on the Ti_6_O_12_H_3_ surface and the edge of the acceptor (see electronic supplementary material, figure S10).

### 3.4. Optoelectronic characteristics of the studied dyes

Concerning TPA-based dyes, we noticed that the functionals Becke's three-parameter Lee–Yang–Parr (B3LYP), half-and-half (BHandHLYP), Coulomb-attenuating method-B3LYP (CAM-B3LYP) and Perdew–Burke–Ernzerhof (PBE1PBE) are applied frequently [69–72]. To evaluate each functional’s overall performance, we computed the absorption spectrum ($\lambda_{\text{max}}$) of RK1 using the functionals B3LYP, BHandHLYP, CAM-B3LYP and PBE1PBE in the gas phase as well as in the two solvents DCM and ethanol. It was found that DCM-B3LYP overestimated the $\lambda_{\text{max}}$, while DCM-CAM-B3LYP and DCM-BHandHLYP underestimated the values in comparison with the experimental results (see electronic supplementary material, table S16). The computed results of PBE1PBE in DCM solvent showed good agreement with the experimental absorption spectrum for RK1 [62] (see electronic supplementary material, table S16). For this reason, we selected PBE1PBE to calculate $\lambda_{\text{max}}$ for the rest of the studied compounds. We used BHandHLYP and PBE1PBE functionals in the gas phase to estimate the $\lambda_{\text{max}}$ values of the dyes@TiO_2_ cluster (see electronic supplementary material, tables S19 and S20). The $\lambda_{\text{max}}$ value computed at DCM-PBE1PBE, that is, 464 nm (see electronic supplementary material, table S16) is in good agreement with the experimental result (470 nm), so we adopted this functional to assess the $\lambda_{\text{max}}$ values of RK2–RK5 as well as dyes@TiO_2_ (see table 3 and electronic supplementary material, table S20). The $\lambda_{\text{max}}$ values of RK1 in ethanol at the BHandHLYP/6–31G** level are 291 and 438 nm for the first peak and second peak, respectively (see electronic supplementary material, table S18). When we compare these values with the experimental data the difference is almost 75 nm for the first peak and 32 nm for the second peak with BHandHLYP. However, in ethanol at PBE1PBE/6–31G**, the $\lambda_{\text{max}}$ values are 336 and 463 nm for the first and second peaks, respectively (see table 3). The difference in $\lambda_{\text{max}}$ for the first peak is reduced as compared with that for the first peak with BHandHLYP, and for the second peak is just 7 nm. Because the experimental $\lambda_{\text{max}}$ of RK1 was measured in ethanol, we used ethanol to calculate $\lambda_{\text{max}}$ by means of PBE1PBE, showing good agreement with experiment (see table 3). The major peak of $\lambda_{\text{max}}$ in the isolated dyes was found at 463, 525, 516, 469 and 534 nm for RK1–RK5, respectively. The $\lambda_{\text{max}}$ bands of the isolated dyes RK2–RK5 are at longer wavelengths (red-shifted) than for RK1. Furthermore, the PBE1PBE in DCM is good at reproducing the experimental results. So, we also calculated the properties in DCM (see electronic supplementary material, tables S16 and S17).

**Table 3.** The computed absorption ($\lambda_{\text{max}}$), light-harvesting efficiency (LHE), major excitations and contribution of RK1–RK5 in ethanol at the TDPBE1PBE/6–31G** level of theory. *f*, oscillator strength; H, HOMO; L, LUMO; exc., excitation. Experimental absorption ($\lambda_{\text{max}}$) is 470 nm for RK1 [62].

dye	ƒ	LHE	exc. *E* (eV)	*$\lambda_{\text{max}}$*(nm)	major exc.	contribution (%)
RK1	0.61	0.752	2.68	463^a^	H→L	15.0
					H→L+1	18.0
					H−1→L	65.0
					H−1→L	13.0
	0.40	0.5100	3.69	336	H→L+2	41.2
					H−3→L	48.0
					H→L+1	12.2
RK2	0.69	0.794	2.36	525	H→L	13.0
					H→L+1	16.0
					H−1→L	64.0
					H−1→L+1	10.0
					H−2→L	20.0
	0.62	0.761	3.33	372	H→L+2	57.0
					H→L+4	13.0
					H−1→L+1	15.3
					H−1→L+2	15.0
					H−2→L+1	32.0
RK3	0.47	0.658	2.40	516	H→L+1	48.0
					H−2→L	50.4
					H−3→L	11.1
	0.71	0.803	3.51	353	H→L+3	66.0
					H→L+4	20.0
RK4	0.45	0.645	2.64	469	H→L+1	21.0
					H−1→L	17.4
					H−2→L	64.4
	1.11	0.923	3.35	370	H→L+2	58.0
					H→L+3	30.2
					H−1→L+2	18.0
					H−2→L+1	11.0
RK5	0.60	0.749	2.32	534	H→L	16.0
					H→L+1	11.0
					H−2→L	63.1
					H−3→L	23.0
	1.40	0.960	3.32	374	H→L+2	19.0
					H→L+3	63.2
					H−1→L+4	15.0

### 3.5. Emission of the studied dyes

The emission performances in two solvents, DCM and ethanol, of isolated RK1 and its derivatives obtained using two functionals TDBHandHLYP and TDPBE1PBE, respectively were considered (see electronic supplementary material, tables S21 and S22). For the predicted emission wavelengths ($\lambda_{\text{max}}^{\text{em}}$) of RK1 at the TDBHandHLYP/6–31G** level, $\lambda_{\text{max}}^{\text{em}}$ for the first and second peaks equals 522 and 315 nm, respectively, and oscillator strength (${ƒ}_{max}^{em}$) for the first and second peaks equals 1.34 and 0.59, respectively. The $\lambda_{\text{max}}^{\text{em}}$ for the first peak of RK1–RK5 is at 522, 503, 490, 515 and 517 nm, respectively. On the other hand, the $\lambda_{\text{max}}^{\text{em}}$ of the second peak for RK1 is at 315 nm while for RK2 it is at 299 nm, which is blue-shifted. Moreover, for RK3–RK5, $\lambda_{\text{max}}^{\text{em}}$ values for the second peaks are red-shifted compared with the parent compound (see electronic supplementary material, table S21). For the predicted wavelengths of RK1 sensitizer at the TDPBE1PBE/6–31G** level, $\lambda_{\text{max}}^{\text{em}}$ for the first and second peaks equals 528 and 380 nm, respectively, and ${ƒ}_{max}^{em}$ equals 0.62 and 0.3, respectively. The predicted $\lambda_{max}^{em}$ in ethanol at the TDPBE1PBE/6–31G** level appeared at longer wavelengths compared with the DCM-TDBHandHLYP/6–31G** level. The $\lambda_{\text{max}}^{\text{em}}$ values for the first peaks of RK1–RK5 are at 528, 571, 561, 577 and 575 nm, respectively, showing red shift in the designed derivatives compared with the parent molecule (see electronic supplementary material, table S22).

### 3.6. Reorganization energy, ionization potential and electron affinity

By using the B3LYP/6–31G** level, the values of charge transport properties of the studied compounds were calculated, that is, adiabatic electronic affinity (EA_a_)/vertical electronic affinity (EA_v_) and adiabatic ionization potential (IP_a_)/vertical ionization potential (IP_v_) (see electronic supplementary material, table S23). Better electron transport performance in organic semiconducting materials is associated with higher EA, whereas better hole charge transport ability is strongly correlated with lower IP. The results show that substituting the TPA and BPA units for H at R1 and/or R2 at the donor side of RK1 increases the EA_a/v_ and decreases the IP_a/v_. It can be seen that the lowest IP_a_ is recorded for RK3 when a BPA unit is substituted at R1/R2 in RK1. Similarly, RK5 records the second lowest IP_a_, where the TPA unit is substituted for H at R1/R2 in RK1. Additionally, substituting the BPA and TPA units at both positions R1 and R2 in RK1 decreases the IP_v_ more than other dyes. It can be observed that substituting the TPA unit at R1 and/or R2 in RK1 enhances the EA_a/v_ compared with RK1. The dyes RK2–RK5 would have better hole and electron injection capabilities than parent dye RK1, as seen by the smaller IP_a/v_ and higher EA_a/v_ values. It is revealed by the lower IP and higher EA that our newly designed dyes might make good p- and n-type compounds [73]. Also, the $\lambda_{\text{h}}$ values of RK1 derivatives are smaller than the $\lambda_{\text{e}}$ values, showing that these compounds would be better hole transfer candidates (see electronic supplementary material, table S23). While several parameters affect DSSC performance, the most essential ones are IP, EA, *λ*_h_ and *λ*_e_. Electronic supplementary material, table S23 shows the IP, EA, *λ*_h_ and *λ*_e_ values computed at the B3LYP/6–31G** level. The λ_h_ and λ_e_ help not only to compute the charge mobility but also to shed light on the DSSC efficiency. It is well known that smaller reorganization energy leads to better charge injection, resulting in increased DSSC efficiency [74]. We provided total polarization from neutral to anion and cation as total reorganization energy (*λ*_total_). We found that the newly designed dyes, except RK3, have smaller internal polarization as compared with the parent compound RK1, which reveals that the designed dyes would have better DSSC efficiency than the parent compound (see electronic supplementary material, table S23).

### 3.7. Chemical reactivity descriptors

The DFT approach provides theoretical parameters pertaining to the characteristics of the new compounds. As an illustration, global chemical reactivity descriptors (GCRD), such as the electrophilicity index (ω), softness (S), absolute electronegativity (χ), chemical potential (μ) and chemical hardness ($\eta_{h}$), can be used to determine the stability and reactivity of molecular structures [75]. Thus, the assumptions of absolute χ and $\eta_{h}$ are entirely consistent with the molecular orbital (MO) theory. Indeed, they appear to supplement it in a way that could be quite beneficial [76]. The chemical hardness $\eta_{h}$ makes it evident that soft materials have a small energy gap between HOMO and LUMO, while hard compounds have a large one [77]. For the dyes RK1–RK5, we calculated the values of S, χ, μ, $\eta_{h}$ and ω. In any two molecules, there will be a partial transfer of electrons from the low *χ* to the high χ molecule. Consequently, these electrons likewise move from high μ to low μ [76]. Moreover, the RK1–RK5 reduced $\eta_{h}$ values indicate that these sensitizers would be more stable. Among all the dyes, RK5 has the lowest value. In our newly designed dyes RK2–RK5, the inclination to provide particles was revealed by the acquired values of *μ*, which showed the donor (D) and acceptor (A) units within the parent compounds. The acquired values of *μ* for RK2–RK5 are smaller than for RK1, which would enhance the stability (see electronic supplementary material, table S24) [75,77,78].

### 3.8. Dye adsorption on TiO_2_ cluster

Electronic supplementary material, figure S11 displays the optimized geometrical structure of the sensitizers adsorbed on the surface of the TiO_2_ cluster. By identifying the most significant bond lengths, bond angles and dihedral angles in electronic supplementary material, table S25, we compare the isolated dyes and dyes@TiO_2_ cluster. Tridentate, bidentate and monodentate are some of the common adsorption types [79,80]. According to earlier research, the bidentate bridging mode is optimal for more stable adsorption, and in our work the dyes RK1–RK5 were adsorbed on the surfaces of the Ti_6_O_12_H_3_ in this manner. Dyes were adsorbed on the titanium atoms after being deprotonated, as shown in electronic supplementary material, figure S12. This process occurred on the surface of the Ti_6_O_12_H_3_.

Following adsorption, there are variations in bond lengths between adsorbed and unbound dyes on the surface of the TiO_2_ cluster, as can be seen from electronic supplementary material, table S25. The C3—O1 bond length of the adsorbed dyes RK1–RK5 is shortened compared with the isolated dyes because the semiconductor surface withdraws charge density towards itself owing to the adsorption and deprotonation processes, which reduces the bond distance. However, the C3—O2 bond length of the adsorbed dyes RK1–RK5 is increased compared with the bond lengths of the isolated dyes RK1–RK5. The C3—O1 and C3—O2 bond lengths are affected more on the acceptor side than the other bond lengths in RK1–RK5 (see electronic supplementary material, table S25). In electronic supplementary material, table S25, the values of the bond distances of Ti1-O1 are smaller as compared with the prior research, which demonstrates that RK1–RK5 dye adsorption on the surface of the TiO_2_ cluster would be stable and strong. Moreover, the bond distance Ti2—O2 is smaller for RK2, RK4 and RK5 and is almost the same for RK1 and RK3 as compared with the prior research, which signifies that adsorption of dyes RK1–RK5 on the surface of Ti_6_O_12_H_3_ would be strong and stable [81]. Furthermore, in comparison with the bond angles of the isolated dyes, O1—C3—O2 is not significantly changed after the adsorption process. However, the O1—C3—C4 bond angle in isolated dyes RK1–RK5 is 112.23° and after adsorption increased for RK1–RK5, that is, to 116.98, 119.09, 117.01, 119.20 and 119.18°, respectively. This indicates that this bond angle is affected more than the others (see electronic supplementary material, table S25). Lastly, the dihedral angle of O2—C3—C4—C6 is more influenced by the acceptor unit than other dihedral angles—following adsorption of dye RK1 on the surface of the TiO_2_ cluster, this angle is greater than the dihedral angle of the unbound dye. The C3—C4—C6—C7 dihedral angle is more affected on the acceptor side than other dihedral angles, while after the adsorption of dye RK3 on the surface of the Ti_6_O_12_H_3_ this angle is decreased (−178.80°) compared with the dihedral angle of the isolated dye RK3 (−179.91°). After the adsorption of dyes RK1–RK5 on the surface of the Ti_6_O_12_H_3_, O1—C3—C4—C5 is more affected on the acceptor side than other dihedral angles; however, it is more influenced in RK2, RK4 and RK5 and is increased in comparison to the dihedral angle of the isolated dyes RK2, RK4 and RK5. In addition, the amount of adsorbed dye on the semiconductor influences the efficiency of the DSSC device. So, the determination and adsorption of dyes on the semiconductor surface are very important. The adsorption energy of RK1 and its derivatives were computed at the B3LYP/6–31G** (LANL2DZ) level. Once the dye is adsorbed to the semiconductor’s surface, the dye’s charge is transferred to the semiconductor. Therefore, we computed geometry to explore and display data, and comprehend their molecular structure. In addition, we computed the adsorption energy of dyes@Ti_6_O_12_H_3_ to predict the stability of the adsorbed dyes. A good adsorption process was helped by choosing the best acceptor unit. Several dyes that use cyanoacrylic acid as an acceptor group were found to undergo *cis*/*trans* photoisomerization when exposed to UV or visible light [82]. The negative values confirm that dye adsorption would be stable on the TiO_2_ (see electronic supplementary material, table S26)

### 3.9. Parameters related to photovoltaic performance of the dye-sensitized solar cell

As shown in electronic supplementary material, table S27, we computed the values of the electron injection and coupling constants for RK1 and the newly designed dyes RK2–RK5. By increasing the donor strength, the values of electron injection and coupling constant are enhanced, as illustrated in the results for dyes RK4 and RK5 in electronic supplementary material, table S27, which shows that the replacement of hydrogen atoms by the TPA moiety at the two positions R1 and/or R2 gave better results than did the BPA moiety. To enhance DSSC efficiency, larger values of the electron coupling constant would be preferable. Furthermore, we observed that TPA group substitution greatly increases the values of the electron injection and coupling constants, indicating that RK4 and RK5 might have superior injection capability.

### 3.10. Power conversion efficiency

Following electronic supplementary material, equation S11, we can consider the photovoltaic parameters short-circuit current density ($J_{\text{SC}}$), open-circuit voltage (*V*_OC_), fill factor (FF) and power conversion efficiency (%η) presented in table 4 and electronic supplementary material, table S28. Table 4 shows the photovoltaic performances of RK1–RK5 at the B3LYP/6–31G** and TDPBE1PBE/6–31G**/6–31G** (Ethanol) levels. We used RK1 as a reference dye, which previously reported an η of 6.04% in metal-free-sensitizer-based DSSCs. The computed value of the efficiency of the DSSC based on RK1 is 6.29%, which is in good agreement with the experimental result. Compared with this computed value for RK1, the DSSCs based on RK2, with one substituting BPA unit at position R1 in RK1 (η = 8.32%), on RK3, with two substituting BPA units at positions R1 and R2 in RK1 (η = 6.60%), and on RK5, with two substituting TPA units at positions R1 and R2 in RK1 (η = 8.05%), show significant improvement in η. However, the DSSC based on RK4, with one substituting TPA unit at position R1 in RK1, shows much lower efficiency (η = 5.36%) compared with the others. These results indicate that modification of the increasing strength of the donor part makes a significant contribution to the enhancement of device performance. In addition, that the DSSC with RK2 exhibits a higher η value compared with the others is due to its $J_{\text{SC}}$. Although the power conversion efficiency of the RK1 device is lower compared with RK2, RK3 and RK5 based DSSCs, the latter exhibits the best performance, indicating that increasing the strength of the donor unit is beneficial to acquire higher η and improved light-harvesting ability. In addition, the photovoltaic performances of the DSSCs based on RK1–RK5 dyes at B3LYP/6–31G** and TDBHandHLYP/6–31G** (DCM) are shown in electronic supplementary material, table S28. The results show the theoretical value of η of the DSSC based on the RK1 dye is 5.79%, which is in good agreement with the experimental data (η = 6.04%). Compared with this predicted value for RK1, the DSSCs based on RK2, with one substituting BPA unit at position R1 in RK1 (η = 7.94%), on RK3, with two substituting BPA units at positions R1 and R2 in RK1 (η = 8.54%), on RK4, with one substituting TPA unit at position R1 in RK1 (η = 7.50 %), and on RK5, with two substituting TPA units at positions R1 and R2 in RK1 (η = 8.24%), show significant enhancement in η. The DSSC based on RK4 dye at B3LYP/6–31G** and TDBHandHLYP/6–31G** (DCM) shows much higher efficiency (η = 7.50%) compared with η = 5.36% at the B3LYP/6–31G** and TDPBE1PBE/6–31G**/6–31G** (Ethanol) levels as, in the prior case, *J*sc is higher, leading to enhanced efficiency.

**Table 4.** Theoretical open-circuit photovoltage (*V*_OC_), short-circuit density (*J*_SC_), and the power conversion efficiency (*η*) at B3LYP/6–31G** and TDPBE1PBE/6–31G** (Ethanol) for RK1 and its derivatives.

dye	*J*_SC_ (mA cm^−2^)	*V*_OC_ (eV)	*η* (%)
RK1	10.15	0.86	6.29
RK2	13.28	0.87	8.32
RK3	10.41	0.88	6.6
RK4	8.65	0.86	5.36
RK5	12.85	0.87	8.05

## 4. Conclusions

The computed geometrical parameters at the B3LYP/6–31G** level are in good agreement with the available crystal data of RK1. We have computed the absorption spectrum ($\lambda_{\text{max}}$) of RK1 using four different functionals, B3LYP, BHandHLYP, CAM-B3LYP and PBE1PBE, in the gas phase as well as in the solvents DCM and ethanol. It was found that the values computed using the PBE1PBE functional in solvents are in good agreement with the experimental data. The ionization potential values of RK2–RK5 are smaller than that of RK1, revealing that the former compounds would easily remove electrons. The negative values of the adsorption energies indicate that the dyes after adsorption on TiO_2_ would be stable. Substituting the BPA moiety for H at R1 in RK1 enhances the efficiency (*η*) to 8.32% compared with RK1 (η = 5.79%). Moreover, substituting TPA moieties at positions R1 and R2 in RK1 boosts the efficiency to 8.05%. Donor strengthening is a suitable molecular design strategy to boost photovoltaic performance by improving DSSC light-harvesting efficiency and enhancing power conversion efficiency.

## Data Availability

The relevant data is uploaded in the Dryad Digital Repository [83]. Supplementary material is available online [84].
